# Correction: Single-cell in vivo imaging of cellular circadian oscillators in zebrafish

**DOI:** 10.1371/journal.pbio.3001382

**Published:** 2021-08-16

**Authors:** Haifang Wang, Zeyong Yang, Xingxing Li, Dengfeng Huang, Shuguang Yu, Jie He, Yuanhai Li, Jun Yan

In the Funding section, the authors omitted a source of funding. The financial disclosure should read:

This work was supported by National Science Foundation for Young Scientists of China grant (No. 31701029) and Natural Science Foundation of Shanghai grant (16ZR1448800) to HW; NSFC-ISF Joint Scientific Research Program grants (31861143035) to JY; Natural Science Foundation of China grants (No. 31571209 to JY and No. 81401279 to ZY); and Shanghai Basic Research Field Project (grant no. 18JC1410100). The funders had no role in study design, data collection and analysis, decision to publish, or preparation of the manuscript.
