# Supplementary material for: Single-cell in vivo imaging of cellular circadian oscillators in zebrafish
Source: PLoS Biol. 2020 Mar 13;18(3):e3000435. doi: 10.1371/journal.pbio.3000435 (PMC7069618; doi:10.1371/journal.pbio.3000435)
Supplement: Attachment — Submitted filename: reply_to_reviewer_final_2_JY_revised_20191119.docx [file pbio.3000435.s039.docx]

**Point-by-point Replies to Reviewers**

The comments from the reviewers are in regular font, and our replies are in italic font.

Reviewers' comments:

Reviewer #1:
The study is novel in terms of approach and in term of findings. A new tool has been developed, using an established clock reporter,  to monitor circadian clock function at a single-cell level in an intact animal (the zebrafish larvae) and to study the ontogeny of the circadian clock. The new finding is that the cellular clocks require light exposure in order to start ticking and this is different than what was previously thought. In addition to these main finding a description of the sequential development of the clock in the brain (starting in the pineal gland) and the two different types of photoreceptor cells with differential clock functions are new and important.
In spite of a very complex subject and complex methodology the manuscript is clear and easy to follow.

Major comments
1. The main finding that light is required for the initiation of cellular clocks is different from what has been previously described and suggested. The groups of Whitmore and Foulkes have shown, in zebrafish cell lines, that a pulse of light synchronizes the period of unsynchronized cellular oscillators. The group of Gothilf, suggested that the same thing may occur in the pineal gland, but have not directly shown this. In fact in the reference used here (ref. 30) is it stated that "It is unknown whether onset reflects the initiation of the oscillator or the synchronization of many, already free running, cellular oscillators. The data presented in this study cannot entirely support or contradict either of these two alternatives."  It seems that the current study solves this question for the pineal cells.  Therefore, I would like to speculate that the differences between the current study and the studies in cell lines reflect cell-type differences. There could be other reasons for this contradiction. In any case, I think it is very important that the authors discuss the reasons for the differences between their findings and earlier studies.

*As pointed out by the reviewer, we also noticed this major difference between our in vivo result and previous results in zebrafish cell lines. By imaging per1-luc transgenic fish, Dekens et al. suggested that cells in the embryos kept under constant environmental conditions may undergo asynchronous oscillations. Ziv et al. also proposed that the expression of aanat2 is asynchronous in individual pineal cells under constant darkness. Carr and Whitmore showed that light-sensitive cells of zebrafish DAP20 cell line kept under long-term darkness became desynchronized and light can synchronize the clocks in these cells. To our surprise, we did not observe asynchronous oscillations of nr1d1:VNP among individual cells in our reporter fish raised under constant darkness. In Dekens et al.’s study, separate zebrafish siblings fixed at CT3 and CT15 respectively under constant dark conditions have an intermediate number of cells expressing per1 at the RNA level. Similarly, Ziv et al. observed an average level of aanat2 in the zebrafish embryos kept in constant darkness compared with the fish kept in light-dark cycle. However, these observations are not the direct evidence for asynchronous oscillations as the gene expression was not traced over time in the same fish. Instead, they can be explained by the highly variable baseline gene expression between single cells within the same fish observed in our study. The difference between our study with Carr and Whitmore’s study may reflect the difference between in vivo vs. in vitro systems. During our time of imaging, zebrafish embryos are undergoing rapid changes of cellular states through differentiation and proliferation, which may preclude the existence of a self-sustained cellular clock. The zebrafish cell line used by Carr and Whitmore is a stable transfected cell line and can harbor free-running autonomous clocks as demonstrated in some stable mammalian cell lines. However, it has also been shown that mammalian iPS cells are non-rhythmic suggesting that the difference of cellular states plays an important role in the state of cellular clocks.*

*As suggested by the reviewer, a deep comparison and discussion of our study and earlier studies have been included in the last paragraph of discussion part.*

2. The authors show that pineal cells with high basal levels of expression have low amplitude rhythms. Could this reflect a technical problem? If fluorescence has reached saturation, it would be impossible to detect rhythms in cells that express high fluorescence levels (rod-like cells in this case). I may be wrong but such saturation appears to occur in some individual traces in figure 4c. Please make sure that these individual cells that reach saturation (if indeed exist) do not affect your results and conclusion regarding the two-cell theory.

*For the pineal imaging, we used a quite low laser power (33.0-35.5mW) to prevent the saturation. No cells have reached saturation in the course of our imaging during development and the 2-h interval time-lapse imaging. Using the data collected every 2 hours for one day, we still find the same result. So we think that this phenomenon is not due to a technical problem. Similar negative correlation between mean transcriptional activity and relative amplitude of circadian oscillations has also been reported in mouse (Westermark, Pål O., 2016 PLOS Genetics). For the cod-like cell imaging dataset, we did observe 5-10% cells reach saturation (Figure 4c). However, this did not affect our conclusion that the baseline level is different between rod-like and non-rod-like cells.*

3. nr1d1 is a clock gene and also a light induced gene (Ben Moshe et al., 2014). Is it reflected in the results? Is it being considered?  *Yes, it is reflected in our result that nr1d1 is a light induced genes (nr1d1-VNP mRNA reaches its peak when the light is on), like per2, cry1b, and other core clock genes. But it is still rhythmic in the first day when the fish was transferred from LD to DD. So nr1d1 reports both circadian and light effects.*
4. 4. I may have missed this. In the in vivo imaging that was done every 12 hours,
   larvae were anesthetized and embedded at each time point.  How was it possible to detect the same cell each time?
   *Answer: Yes, imaging was done every 12 hours for the same fish after fish larvae were anesthetized and embedded at each time point. Because we have single-cell resolution in our reporter, each single cell was traced by aligning 3D images at different time by an image alignment tool: CMTK toolkit. After image registration, the same single cell of the same fish can then be identified across different time points. We included supplementary movies to show the 3D images after registration.*

Minor comments:

Given the main point of the paper is the cellular clocks, consider adding 'cellular' to the title: "Single-cell in vivo imaging reveals light-initiated cellular circadian oscillators in zebrafish"
*As suggested by the reviewer and also to reflect our paper as a “Resource and Method”, we have changed the title to "Single-cell in vivo imaging of cellular circadian oscillators in zebrafish".*

Results, 'Characterization of nr1d1:VNP expressing cells'. It is important to emphasize the high levels of nr1d1 mRNA in the pineal cells (fig 2b). It strgenthens the finding using the transgene (fig 1c and fig 1e).
*As suggested by the reviewer, the high expression of nr1d1:VNP in the pineal gland were emphasized in the ' Characterization of nr1d1:VNP expressing cells' section.*

Results, second paragraph. It is stated that "both genes showed higher expression level at the dawn than dusk over days (Fig. 1b)" but in figure 1b it seems that the samples were taken in mid-day and mid night.

*Answer: Yes, this was a mistake. Fig 1b has been modified accordingly.*

3. The use of 'rod' and 'cone' cells is confusing because rods and cones are retinal cells. I suggest naming them 'rod-like' and 'cone-like'. Also, the term 'rod fish', although clear to me, is not appropriate.
   *As suggested by the reviewer, 'rod' and 'cone' cells have been replaced by 'rod-like' and 'cone-like cells. And ‘rod fish’ were changed to Tg(xops:nfsB-mCherry) fish.*

   What is the rod marker xops?  I cannot find the primers used for xops (materials and methods) in the zf genome nor in frog genomes

*The xops is Xenopus rhodopsin gene (GenBank: U23808.2). The xops promoter (1370 bp) was amplified from pFIN-XOPS-tdTOMP (Semple-Rowland SL et al., 2010, addgene #44359) using forward (5’-tatagggcgaattggGGCCGCAGATCTTTATACATTGC-3’) and reverse (5’-CCGGTGGATCCCAAACCCTCGAGATCCCTAGAAGCCTGTGAT-3’) primers containing homogenous recombination sites, respectively.*

Discussion 2nd paragraph, "It was believed that physiological and behavioral rhythms in zebrafish appear during the early stages of zebrafish development [8]."  This is a vague sentence.  It is not clear what is 'early stages', I guess it refers to larval stages because behavioral rhythms are not seen in early stages of embryogenesis.  If indeed the meaning is larval stages than the frase 'it is belived' is not accurate – it was shown. I suggest to change this sentence to " It has been shown that physiological and behavioral rhythms in zebrafish appear during the larval stages of zebrafish" Also, maybe a better reference than no. 8.
*As suggested by the reviewer, the sentence was changed to " It has been shown that physiological and behavioral rhythms in zebrafish appear during the larval stages of zebrafish". New references were also included here.*

Results, first paragraph. Please indicate how were the plasmids of bmal1a/bmal2/per2/nr1d1:TGFPD1 screened. Was that performed by stable or transient expression? If stable, in which generation?

*We screened all the plasmids by injecting the plasmids into the embryos at one-cell stage. Then we checked the expression of fluorescent proteins in the F1 generation fish. We found only nr1d1:VNP plasmid was expressed in the CNS including the pineal gland in F1 fish. So we only construct the nr1d1:VNP fish line afterwards as a stable fish line after three generation.*

Results, second paragraph. The presence of CRX/OTX binding sites is probably the reason for the enhanced pineal expression. Please mention this.
*Yes, indeed. This sentence has been added to the discussion.*

Results, second paragraph, " scRNA-seq data also suggested that nr1d1:VNP is expressed in proliferative cells". Please emphasize that this is in the pineal gland, and at what age? This is important because it means that photoreceptors are still proliferating.
*As requested, we have emphasized that we found co-labeling of VNP and DsRed in pineal at 5.5dpf. This indicated that a small number of photoreceptors are still proliferating at 5dpf. It may also be due to nr1d1 expression in other cell types within pineal gland, such as glia cells, which also express high levels of Her4.*

Minor editorial points

Page 6, 2nd paragraph, epidemis should be epidermis.
*Modified.*
Page 7. The word 'the' is missing: "…we found that VNP positive cells within the pineal gland display"
*Modified.*

Page 8, bottom "We also observed that the cluster with higher baseline level (cluster 1) also showed".  I think there is a confusion and this should be cluster 2. Please check.
*Yes, it should be cluster 2.*

*Modified.*

Page 9, "signals in zebrafish larvae raised under the constant…"  remove 'the'.
*Modified.*

Figure 2c and 2d should be separated. Its confusing
*Modified.*

Methods, 'in vivo imaging', bottom. Starting with "Two fish under light-dark (LD)….". It will be informative to add figure numbers.
*Figure numbers were added.*

Methods, Single cell RNA-seq and data analysis. This is written like someone's protocol.

*Yes, we have have modified this part of methods to make it more succinct.*
Figure 1c. need orientation

*Yes, we have modified the legend for Figure 1c to make it more clear.*

Figure 1 legends, "The putative RRE (Nr1d1/2 binding site), E-box (Bmal1/Clock
binding site), Crx, Otx5 and Crx/Otx5 binding sites were indicated in blue rectangle,
red oval, green oval, yellow rectangle, and dark green oval respectively". There is a mistake in the order. And there are two oval greens.
*Yes, this was a mistake and has been modified. The two green ovals are different. One is light green and the other is dark green.*
Figure 2 legends, (a) should be "t-SNE visualization of brain cell clusters…"
*Modified.*
Figure 2 legends, (b). please add: "note the highest levels in the pineal gland"
*Added.*
--

Reviewer #2:

The paper by Wang et al., addresses the question of whether cellular clocks exist and function spontaneously from the initial cell divisions of embryogenesis or alternatively whether they are initialised/activated by an environmental signal (light) during development. To address this question Wang et al., have developed a reporter line nr1d1:VNP in transgenic zebrafish to report single cell dynamics of a clock driven reporter. The authors use this clock reporter line to measure single cell dynamics in all cells of the embryonic zebrafish brain/pineal from 3.5 to 6.5 dpf. They try to separate circadian and developmental dynamics.

The authors are correct in that zebrafish are the perfect preparation to address this question. In addition, this issue has been debated greatly in the literature, with some groups promoting the spontaneous and synchronous start of the clock to those that suggest a spontaneous beginning to oscillations, but with a need for an entrainment stimulus (light) to synchronize cells. The authors are correct in that the only way to approach this problem is by single cell imaging of clock function in the living embryo, and they have generated a superb tool to test this hypothesis.

My main problem is that I don’t feel they have succeeded in answering this question yet by the nature in which they have performed their imaging experiments.

The authors performed imaging experiments at set intervals rather than performing continuous (or relatively continuous) imaging on identified cells. Initially only looking at two time points per day, but then every two hours over one cycle in constant darkness. Two time points is not sufficient resolution to look for possible phase differences between oscillators. And the way that the imaging is performed is akin or similar to typical molecular sampling or performing in situ hybridization experiments.

There are two possible options in the DD experiment. a) either the clock is not oscillating in each cell, which will produce a “flat-line” in expression as seen or b) the cells are oscillating in DD but with completely different and dispersed phases, which by sampling in this way would also produce a “flat” data set, but with significant error bars, reflecting the diverse phases of the cells. Both biological states give the same apparent outcome.

In these imaging experiments, it is essential that identified cells are followed dynamically, such that one can follow the changes in expression within an individual “known” cell. In this way, either the expression levels will be flat in a given cell or may oscillate, but with random phases in the population. One cannot randomly pool the data from the population imaging.

*There is a misunderstanding here. In fact, we did exactly as reviewer suggested. All identified cells were followed dynamically at different time points in the same fish and the changes in expression within an individual “known” cell have been followed. This was accomplished by image registration, aligning 3D single-cell image of the same fish over time so that we can trace fluorescence changes for the same cell. For Fig 1d, Fig 3, Fig4, and Fig5, each thin line represents the tracing result of one single cell, and the thick line represents the mean of the cell population. So at single cell level, we did not observe asynchronous oscillations of nr1d1:VNP in our reporter fish raised under constant darkness suggesting that cellular oscillators were not initiated without environmental stimuli. We performed time-series imaging every two hours per day for both the fish raised under constant darkness and under light-dark condition. According to that data shown in Fig 4, we found that the peak time of the nr1d1:VNP were at the dusk. So for the subsequent development study, we imaged the fish at the dawn and the dusk, respectively.*

*And we agreed that imaging at higher temporal resolution will enhanced our result, so in this version of the paper, we tried to image the fish every one hour for one day (Fig.1f & g), we found that 39 out of 48 cells showed oscillating using JTKcycle adjusted  P<0.05 and absolute amplitude of oscillation >100 as the cutoff. This demonstrated that we can do continuous imaging using this system.*

A less important, but significant point. When does the rhythm in nr1d1 begin in development, at a global level on an LD cycle? Multiple papers have shown that the period gene rhythm is apparent globally somewhere on the second day of development – 4 hour or so onwards. The imaging data in this study is from day 3.5 onwards, when it is known that the clock is running. If the nr1di rhythm starts latter in development, i.e. the second clock loop develops more slowly than the primary loop, then sadly this reporter is not optimal for studying when or how the clock begins. A simple in situ or qPCR data set would be very useful to address this.

*As suggested by the reviewer, we performed qPCR of zebrafish larvae at different early embryonic stages to address this question. Our qPCR data (Fig S1a) have shown that nr1d1 mRNA in fact starts oscillating very early from the second day of development, as early as per1b gene. The developmental profile of nr1d1 mRNA is highly correlated with per1b. Therefore, nr1d1 reporter just like per1b is optimal for studying when or how the clock begins. Furthermore, we started imaging from 3.5dpf because the fluorescence signal is too low to be detected before 3.5dpf using the same laser power as we used when we imaging the fish after 3.5dpf. In this experiment, we did not use high laser power because it may hurt the larvae fish and also the fluorescence in many cells can reach saturation afterwards.*
There are number of other minor comments to make. However, until the imaging is repeated with identified cells, these are of little significance. The authors have made a very powerful circadian tool and I am sure they will use it to generate a great deal of new, insightful data. However, at this point I do not think that they are quite there yet. Though many of their results are interesting.

Regarding the tissue specific nature of their reporter. This makes this a powerful tool to study the pineal gland, and the establishment of rhythms later in other brain regions, but perhaps by using a more ubiquitous promoter they might be able to follow oscillations is earlier stages of development.

--

Reviewer #3:
In this study, Wang et al., examined whether circadian oscillation of the pineal gland in zebrafish is stopped or desynchronized during development without LD cycles. They generated a transgenic zebrafish line carrying a destabilized fluorescent protein to monitor one of clock genes, nr1d1, at the single cell level using a two photon microscope. Since single cell time-lapse imaging of clock gene expression in vivo have never been performed, it still unclear how clock gene expression occurs under DD in zebrafish. By using this method, they confirmed Nr1d1 expression rhythms in the pineal gland and other areas under LD cycle. They found two different populations in the pineal gland. One showed rapid increase in the baseline level while the other showed robust circadian oscillations, which were corresponding to rod and non-rod cells, respectively. Importantly they found that nr1d1 expression in the pineal gland did not show circadian oscillations under constant darkness. From
these results, they concluded that light exposure in early clock development initialized cellular clocks. The hypothesis is interesting and optical imaging of single cells in vivo is fascinating. However, they have not shown enough data and analysis to support their conclusion. There are some concerns for the authors to address.


Major concerns
1. The authors concluded that cellular circadian oscillations were not initiated without environmental stimuli (also the title of the manuscript). This is too strong a statement from the results. Previous studies suggested that circadian rhythms (per expression) were desynchronized in the embryos under DD. The discrepancy between these reports and the present study might be due to different clock genes. There are multiple circadian feedback loops, such as Per/Cry loop and Bmal1/Clock/Reverb-a loop. The author measured only nr1d1 expression, but if they measure per expression in single cells, circadian rhythms might be detected under DD. Without this data, it is difficult to conclude that “circadian oscillations” were not initiated under DD. In addition, the authors need to deeply discuss discrepancy between previous studies (Denkens et al. 2008; Carr et al. 2005) and the present study.
*As suggested by this and the first reviewer, we also noticed this major difference between our in vivo result and previous results of desynchronized oscillations in zebrafish cell lines under DD. We believe that the difference is due to the difference of in vitro vs. in vivo systems. During our time of imaging, zebrafish embryos are undergoing rapid changes of cellular states through differentiation and proliferation, which may preclude the existence of a self-sustained cellular clock. The zebrafish cell lines used by Whitmore and Foulkes’ groups are stable transfected cell lines (DAP20) and can harbor free-running autonomous clocks like in some stable mammalian cell lines. However, it has also been shown that mammalian iPS cells are non-rhythmic. Therefore, the difference of cellular states plays an important role in the state of cellular clocks. As suggested by the reviewer, a deep comparison and discussion of our study and earlier studies have been included in the last paragraph of discussion part.*

*To address whether nr1d1 is appropriate to report circadian clock development as compared to other clock genes, we performed qPCR of zebrafish larvae at different early embryonic stages. Our qPCR data (Fig S1a) have shown that nr1d1 mRNA in fact starts oscillating very early from the second day of development, as early as per1b gene. The developmental profile of nr1d1 mRNA is highly correlated with per1b. Delaunay et al.’s whole mount ISH (Delaunay et al. 2000 Figure 1) data also showed that nr1d1 start oscillating at 2dpf in the pineal gland, as early as per1b gene. Therefore, nr1d1 reporter is appropriate for studying the early development of circadian clock.*

2. The authors measured nr1d1 expression every two hours from 5.0 to 6.0 dpf under DD and concluded that circadian rhythms were not detected at the single cell level (Figure 5i and k). Please clearly describe how the significance of circadian rhythms was analyzed. Cosiner method was used for rhythm analysis or other methods? This is a very important point in this paper, because low amplitude oscillation and no rhythmicity are totally different. The authors need to evaluate circadian rhythmicity in single cells by using several methods. In addition, low resolution time point data might result in false negative. The authors also need to discuss this point.
*The circadian phase and amplitude of each cell were calculated by fitting the mean intensity of each cell to cosine functions with 24 hours’ period and shifting phases as described in our previously paper (Yan et al. 2008). The cells with cosine fitting P<0.05 and relative oscillating amplitude>0.05 were defined as oscillating cells. As suggested by the reviewer, in this revision, we also applied JTKcycle (from MetaCycle package in R), a widely accepted package to analyze the significance of circadian oscillation, JTKcycle p-value<0.05 and absolute amplitude>100 were used as the cutoff to define rhythmic cells. We can see that the result are quite consistent with our previous cosine fitting method as shown in Fig 5j,k (JTKcycle) and Fig S4(cosine fitting).*

*We agreed that we cannot completely rule out the possibility of low amplitude oscillations, since we use amplitude as a cutoff to select the oscillating cells. So we have changed our statement to ‘single-cell oscillations were dramatically dampened in animals raised under constant darkness’.*

*Furthermore, we have increased temporal resolution by imaging the fish every 1 hour for one day (Fig 1f & g) and both cosine fitting and JTKcyle were applied to analyze the data.*

Minor concerns
1. The authors used cosine function with 24 hours period to determine circadian phase and amplitude (p18). Since circadian period and trend is different cell by cell, they should add two more parameters “period” and “trend” to precisely determine phase and amplitude.
*As suggested by the reviewer, in this revision we also applied JTKcycle from MetaCycle package to analyze the significance of circadian rhythm. ‘period’ of each single cell were estimated by JTKcycle. For the developmental data from 3.5dpf to 6.5dpf, we decomposed the expression to development and circadian components, the development component here represents the trend over time.*

2. In this research, analysis of circadian rhythms in single cell level is critical. Please show nr1d1 expression data of individual cells under DD with cosine fitting data in Figure 5i similar to Figure 4c. Also it is better to show raw fluorescence data in Figure 5i to be able to directly compare LD and DD conditions.
*As suggested by the reviewer, Fig 5i were modified to show the raw fluorescence intensity. We also added the expression pattern of each DD cells in Fig S4c.*
3. The author found two clusters in the pineal gland (Figure 4e-h). These two clusters were observed under constant darkness? It seems not to be observed two clusters under DD (Figure 5i). If not, these clusters were driven by light dark cycles? Furthermore, please discuss physiological roles of these two populations in the pineal gland.
*The two clusters in pineal gland were observed under LD condition but only one cluster was observed under constant darkness. Our explanation is that the cells of fish raised under constant DD from birth do not show significant circadian oscillations and therefore only exhibit development trends.*

4. P5, 2nd para. The authors mentioned “both genes showed higher expression level at the dawn than dusk over days (Fig. 1b)”. But they measured these gene expression rhythms during middle of day and night, so dawn and dusk is not appropriate here.
*This was a mistake. Fig 1b has been modified.*

5. P7, 1st para. The authors compared nr1d1:VNP and aanat2:mRFP, xops:nfsB-mCherry, lws2:nfsB-mCherry, or her4:Dsred reporter. Some VNP positive neurons seem not to express mRFP in Figure 2c. Please indicate the number of cells, percentage of overlap, and the number of fish. Especially, xops:nfsB-mCherry data is important because they separated rod and non-rod cells using the reporter in Figure 3.
*As suggested, the number and percentage of overlap cells were indicated in the text. 94% nr1d1:VNP signals were overlapped with the aanat2:mRFP signals.*
6. Figure 3e and d; p7-8. The authors used fitting analysis for the data obtained from 3.5 to 6.5 dpf. They mentioned that “rod cells have a faster rate of developmental increase in nr1d1:VNP signals (A) but lower amplitude of circadian oscillation (B) than non-rod cells (Fig. 3f-h)”. If the data is lineally increased, (A) can be indicated as a developmental increase. But in the case of rod cells, the data seem not linear, so (A) can not simply reflect a developmental increase. They also described (B) as amplitude of circadian rhythms. However, the data were obtained from two points per day. If the circadian phase is shifted, it is plausible that (B) became small value. 
*We agreed that it is hard to define the circadian phase using only two time points. But it is clear that rod-like cells have higher baseline level expression than non-rod-like cells regardless which methods we used for data fitting. So in this revision, we modified our statement to ‘we found that rod-like cells have higher level of baseline expression than non-rod-like cells’*.

7. P9, 2nd para. It is difficult to say “the circadian expression had been absent in DD cells” from two points per day data.
*As suggested, it has been changed to ‘no obvious oscillations were observed in DD cells’.*
8. P11, 2nd para. Dekens et al. did not use per1-luc imaging.
*This is a mistake. It has been modified.*
9. P15, 1st para. Laser power should be described like “W/mm2” not “%”.

*Yes, we have changed the units from “%” to “W”.*
10. P15, 1st para. Please indicate number of fish they used in figure legends to easily understand.

*As suggested, we have included fish number in the figure legends.*
11. P15, 1st para. “Three fish under LD_LD condition and Three fish under LD_DD condition were….”. Second “three” should not be capital.
*Yes, it has been modified.*
12. Figure 2b and e. What is the y axis?
*The y axis denotes the normalized expression value.*

13. Figure 3b. There is no information about green circles in the pictures.
*Yes, we forgot to mention that the green circle represents the representative cell highlighted in Fig. 3c (green line) and Fig. 3d.*
14. P18. Please describe t-SNE analysis in detail.
*As suggested, the detailed methods for t-SNE analysis were added.*
--

Reviewer #4:

Summary: The manuscript authored by Wang et al described beautiful recordings on how the circadian clock kicks off during the development and how it maintains after the maturation in the pineal gland, a master organ of the zebra fish clock. Backed with a state-of-the-art technology of single cell-sequencing, the authors believed that this reporter fish line can be used in a variety of stimulation conditions where the cellular clock can be real-timely monitored in vivo. This reviewer support its publication after the revision.

Major concerns:
1. The reporter line was generated by a transgenic technology rather than a site-specific knockin strategy. Even though the authors spent quite a bit space of the ms to explain/ratify the faithfulness of the reporter with the endogenous gene expression, the conclusion of each single experiment ought to be drawn in cautious. For example, the data of tracing the dynamics of nr1d1 (i.e. the clock) in each particular cell are more convincing than the comparison of among them. You can never rule out the position effects of the reporter insertion. After all, the authors, and probably people in the fish clock field, do not know how the ZfP2 regulates nr1d1 expression while nevertheless it still sits in the transgene to keep the function of ZfP1 intact (Fig1a). The populational comparison of reporter signal vs gene expression cannot be extended to single cell level. Of course this reviewer doesn't mean to assert that the reporter is useless or wrong, but it may just like the
Per1-luc transgenic vs PER2::LUC knockin reporters in mammalian clock field, the limitation of this reporter should be addressed in the discussion.

*Thanks for pointing this out! Yes, we agree with the reviewer that this transgenic line has its limitations such as the positional effect of insertion and fish-to-fish variability. By performing time-series imaging on a number of different fish, we have found similar trend at single-cell level indicating our results are not due to individual difference of transgene. We have now discussed about the caveat of using transgenic fish in the first paragraph of discussion part.*

2. A second major concern is that the Fig4 and 5 seem to be separated, and neither were fully investigated. This reviewer possesses a feeling that the authors has raised some interesting questions based on their observations, but stop short of answering them.  However, if this is the "Methods and Resource" section of the journal designed for, I'm OK with that.

*We acknowledged that by using the temporal resolution of our current imaging set up, we can not complete rule out low-amplitude oscillations in some pineal cells. To reflect this limitation and also conform with “Methods and Resource” style of the journal, we tuned down out our conclusion and modified the title as "Single-cell in vivo imaging of cellular circadian oscillators in zebrafish".*

3. The authors summarized in the abstract that "light exposure in early clock development initializes cellular clocks rather than synchronizes existing individual oscillators". While in the main text and figure legend, they claimed that "Light-Dark cycle is essential to synchronous onset of nr1d1 oscillation". Please reconcile them.
*Yes, we changed it to “light exposure in early clock development is necessary for high-amplitude oscillations in single-cell oscillators”.*
Minor concerns:
There are a few typos/mispresentations in the ms. For instances: Fig1b, are the blue/red lines in right phase with x-axis? Figure legends Fig3, there are two (g) (page27).

*Yes, they have been modified.*
