# Supplementary material for: Single-cell in vivo imaging of cellular circadian oscillators in zebrafish
Source: PLoS Biol. 2020 Mar 13;18(3):e3000435. doi: 10.1371/journal.pbio.3000435 (PMC7069618; doi:10.1371/journal.pbio.3000435)
Supplement: Attachment — Submitted filename: reply_JY.docx [file pbio.3000435.s040.docx]

**Point-by-point Replies to Reviewers**

The comments from the reviewers are in regular font, and our replies are in italic font.

Reviewers' comments:

Reviewer #1:
Thank you for addressing all my concerns adequately

--

Reviewer #2:

The revised manuscript PB-D-19-02041R2, by Wang et al, represents a significant improvement from the initial submission. Aspects of the imaging approach have been clarified and the added experiments (qPCR especially) address many of the key biological questions. I believe that this manuscript is now acceptable and worthy of publication in PLOS Biology. The study represents a significant technical achievement and generates biological data that is both interesting and intellectually stimulating. These data should be "seen" by the wider scientific community.

The more one thinks about the data presented the more interesting it becomes and consequently the more questions it generates. But these are for future studies. One comment the authors may wish to add to their discussion is that all clock studies in zebrafish have so far focused on one of the two (or possibly three) feedback loops that make up the circadian clock system. The data in this study is believable, but it is possible that the rapid dampening in DD is something specific to the "second" loop. And the classic clock/bmal - cry/per loop might behave differently. This would explain all of the differences seen between the various zebrafish groups studying this phenomenon. It is an interesting possibility that light input, as well as developmental timing, might vary across the feedback loops…..or the clock itself might develop differentially. The future will tell.

*Thanks for pointing this out! Yes, we agree with the reviewer that the rapid dampening in DD may be specific to the “second” loop. We have added this comment to the last paragraph of discussion.*

Congratulations to the authors on an excellent study.

--

Reviewer #3:

In the revised manuscript, Wang et al. provided supplemental analysis and data to address the previous comments. I remain supportive of this work, but there also remain a few issues which I respectfully disagree.

As I mentioned in the previous comments, low time resolution might result in false negative. The authors performed imaging with high time resolution (every one hour) at 5.0 to 6.0 dpf under LD (Fig 1g). But important point is DD condition. If they include LD data, I recommend them to show one hour data at DD condition to compare LD and DD condition.

*As requested by the reviewer, we now have also included the data under DD in higher time resolution (every 1 hour) (S4d-S4g Fig). As one can see from the result, the circadian oscillations of single cells were again dramatically dampened in DD cells, consistent with the data collected every 2 hours.*

The authors provided expression pattern of each DD cells in Fig S4c as I suggested. However many cells seem to be rhythmic. It is confusing, because they concluded that single-cell oscillations were dramatically dampened in animals raised under constant darkness. Please show the single cell data obtained from DD.

*This is mainly because DD data and LD data were not plotted on the same scale. We have revised Fig S4c so that DD data are on same scale as LD data. Now it is clear that single-cell oscillations of DD cells were dramatically dampened compared with LD cells.*

Fig 5j,k and Fig S4a,b are same data. Since the author performed JTK cycle in Fig.5j,k and cos fitting in Fig S4a,b, the results must not be the same. Please check the original results.

*Sorry, this was our mistake. In this revision, Fig S4a,b have been replaced by the cosine fitting result.*

P13 The author described as “However, it has also been shown that mammalian iPS cells are non-rhythmic…”. I recommend them to add ES cells which is also the non-rhythmic case.

*Yes, ES cells have been added.*

As I mentioned in the previous comments, Per oscillation might be detected in single cell level under DD, even though nr1d1 expression rhythm was dampened. The authors showed nr1d1 and per1b expression rhythms under LD using qPCR data, but it could not answer this question. In addition, circadian amplitude of per1b rhythms was high as compare to that of nrldl. Without a recording of single cell level of Per expression under DD, it is difficult to answer. However it might be beyond the scope of this study. Instead of doing this experiment, please discuss this issue in the manuscript.   
*As also pointed out by Reviewer #2, we agree with this reviewer that the rapid dampening in DD may be specific to the “second” loop. We have added this comment to the last paragraph of discussion. It is certainly worthwhile to compare the single cell expression of nr1d1 and per gene during zebrafish development. Although we have not succeeded in generating a transgenic per-VNP reporter line in this study, it leaves the door open in the future work to quantify the single cell expression of per gene.*

As I mentioned previous comments, I suggested putting two parameters “period” and “trend” into cos fitting in two hours data (one hour data also). Because fluorescence is increased during development and period would be different cell by cell, if they use add two parameters, quality of the fitting results could be increased. I assume that some non-rhythmic neurons might be rhythmic.

*Please note that the analysis of “period” and “trend” is only applicable for circadian time-series data over multiple days. The estimate of “period” will not be accurate for one-day time-series data. Within one day, the predominant effect is circadian effect, while the development trend can only be detected at longer time scale over multiple days. When we analyzed the developmental data from 3.5dpf to 6.5dpf, we decomposed the expression to development and circadian components in which the development component represents “trend” over time. However, when we analyzed the one-day data at high temporal resolution, applying “detrend” operation to the one-day data can artificially diminish the circadian profile. That is why we used “detrend” only to multi-day data but not for one-day data in this study.*

--

Reviewer #4:
The revised version has addressed all my concerns. Therefore, I support its publication asap.
